# Defective Articulation

**Published:** 1889-03-02

**Authors:** 


### DEFECTIVE ARTICULATION.

Mr. William Van Praagh has reprinted from the "Transactions of the Odontological Society" an essay on "Defective Articulation resulting from Cleft Palate." The author states that "with scientific and judicious treatment sufferers from cleft palate and hare-lip of all degrees of severity can be taught to speak in a perfectly intelligible manner." To attain this, the adoption of a "gymnastic practice" is advised. But the gymnastics do not consist in exercising the limbs, being confined to certain movements of the jaw and tongue, which are best performed before a glass. Skill, patience, and enthusiasm on the part of the teacher, and perseverance on the part of the patient will, it is asserted, work wonders. We are not, however, informed as to the precise movements to be made, nor the length of time required to produce results, which presumably must depend on the degree of malformation present; but so long as the malformation, which is the cause of the defective speech, remains, it may be doubted if very great benefit will result from jaw and tongue "gymnastics."

